# Neutralization of SARS-CoV-2 Variants by mRNA and Adenoviral Vector Vaccine-Elicited Antibodies

**DOI:** 10.3389/fimmu.2022.797589

**Published:** 2022-03-08

**Authors:** Takuya Tada, Hao Zhou, Marie I. Samanovic, Belinda M. Dcosta, Amber Cornelius, Ramin S. Herati, Mark J. Mulligan, Nathaniel R. Landau

**Affiliations:** ^1^Department of Microbiology, NYU Grossman School of Medicine, New York, NY, United States; ^2^Department of Medicine, NYU Grossman School of Medicine, New York, NY, United States; ^3^NYU Langone Vaccine Center, NYU Grossman School of Medicine, New York, NY, United States

**Keywords:** SARS-CoV-2 variant, Delta, Delta plus, Lambda, BNT162b2, mRNA-1273, Ad26.COV2.S, Neutralization

## Abstract

The increasing prevalence of SARS-CoV-2 variants has raised concerns regarding possible decreases in vaccine effectiveness. Here, neutralizing antibody titers elicited by mRNA-based and adenoviral vector-based vaccines against variant pseudotyped viruses were measured. BNT162b2 and mRNA-1273-elicited antibodies showed modest neutralization resistance against Beta, Delta, Delta plus and Lambda variants whereas Ad26.COV2.S-elicited antibodies from a significant fraction of vaccinated individuals had less neutralizing titer (IC_50_ <50). The data underscore the importance of surveillance for breakthrough infections that result in severe COVID-19 and suggest a potential benefit by second immunization following Ad26.COV2.S to increase protection from current and future variants.

## Introduction

Severe acute respiratory syndrome coronavirus 2 (SARS-CoV-2) vaccines from two vaccine platforms have been granted U.S. Food and Drug Administration (FDA) Emergency Use Authorization: mRNA-based (Pfizer and Moderna) and adenoviral vector-based [Johnson & Johnson (J&J)], all of which have been shown to be highly effective. The mRNA-based vaccines were 94-95% effective in preventing COVID-19 (1) whereas the adenoviral vector-based J&J vaccine had 66.9% efficacy in preventing moderate to severe disease (2). However, the ongoing emergence of highly transmissible variants with mutations in the spike protein raises concerns regarding possible decreases in vaccine effectiveness due to spike protein antigenic variability.

SARS-CoV-2 variants have been classified by the World Health Organization (WHO) based on increased transmissibility and/or pathogenicity as variants of concern (VOC; Alpha (B.1.1.7), Beta (B.1.351), Gamma (B.1.1.248), Delta (B.1.617.2) and variants of interest (VOI; Epsilon (B.1.427/B.1.429), Iota (B.1.526), and Delta plus (AY.1) and Lambda (C.37) (3). The increased transmissibility and/or pathogenicity of the variants is due, at least in part, to mutations in the spike protein RBD that increase its affinity for ACE2 on target cells. Mutations in the Beta, Gamma and Delta variant spike RBDs have been shown to cause partial resistance to neutralization by the serum antibodies of vaccinated and convalescent individuals and therapeutic monoclonal antibodies (4–11).

This study measured the neutralization titers of serum antibodies from individuals immunized with three U.S. FDA Emergency use authorization vaccines (BNT162b2, mRNA-1273 and Ad26.COV2.S) against viruses with the VOC and Lambda spike proteins. The study groups were controlled for age, clinical co-morbidity, history of pre-vaccination infection and sera were collected on similar days post-vaccination. The results demonstrate a high level of cross-neutralization by antibodies elicited by BNT162b2 and mRNA-1273 on the variants but significantly decreased neutralization by those elicited by the single dose Ad26.COV2.S.

## Materials and Methods

### Clinical Study

Convalescent sera were collected 32-57 days post-symptom onset. For the early time-point, BNT162b2 and Moderna-vaccinated sera were collected on day 28 and 35, respectively, 7 days post-second immunization. For the later time-point, BNT162b2-vaccinated sera were collected 52-110 (mean 90) day post-second immunization and mRNA-1273-vaccinated sera were collected 44-105 (mean 80) days post-second immunization. Ad26.COV2.S-vaccinated sera were collected 57-115 (mean 82) days post-immunization (**Supplementary Table 2**). The clinical study was conducted at the NYU Vaccine Center with participant’s written consent under IRB-approved protocols (18-02035 and 18-02037). REGN10933 and REGN10987 were generated as previously described (7).

### Plasmids

pLenti.GFP.NLuc is based on pLenti.CMV.GFP.puro containing a GFP/nanoluciferase cassette separated by a picornavirus P2A self-processing amino acid motif cloned into the BamH-I and Sal-I sites (Addgene plasmid #17448, provided by Eric Campeau and Paul Kaufman). pcCOV2.Δ19S is based on pCDNA6 in which the CMV promoter drives transcription of a synthetic, codon-optimized SARS-CoV-2 spike gene based on Wuhan-Hu-1/2019 with a termination codon at position 1255 that deletes the carboxy-terminal 19 amino acids (12). Spike mutations were introduced into pcCOV2.Δ19.D614GS by overlap extension PCR and confirmed by DNA nucleotide sequencing. Plasmids used in the production of lentiviral pseudotypes (pMDL and HIV-1 Rev expression vector pRSV.Rev) have been previously described (12). sACE2-Nluc was introduced into pcDNA6 by overlap extension PCR and confirmed by DNA nucleotide sequencing.

### Cells

HEK293T cells were cultured in Dulbecco’s modified Eagle medium (DMEM) supplemented with 10% fetal bovine serum (FBS) and 1% penicillin/streptomycin (P/S) at 37°C in 5% CO_2_. ACE2.293T are clonal cell-lines that stably express a transfected human ACE2. The cells were maintained in DMEM/1 μg/ml puromycin/10% FBS/1% P/S.

### SARS-CoV-2 Spike Lentiviral Pseudotypes

Lentiviruses pseudotyped by variant SARS-CoV-2 spikes were produced as previously reported (12). Briefly, pseudotyped virus stocks were generated by cotransfection of 293T cells with pMDL, pLenti.GFP-NLuc, pcCoV2.S-Δ19 and pRSV.Rev by calcium phosphate transfection. After 2 days of transfection, viruses were concentrated by ultracentrifugation. Virus titer was normalized for reverse transcriptase (RT) activity. Neutralization titers of sera, monoclonal antibody and soluble ACE2 (sACE2) were determined as previously described (12). Briefly, serially diluted soluble ACE2 protein was incubated with pseudotyped virus for 1 hour at room temperature and added to ACE2.293T cells. The luminescence was measured in an Envision 2103 microplate luminometer (PerkinElmer).

### SARS-CoV-2 Pseudotypes Infectivity Assay

To determine neutralizing antibody titers, sera or monoclonal antibodies were serially diluted 2-fold and then incubated with pseudotyped virus (approximately 2.5 X 10^7^ cps) for 30 minutes at room temperature and then added to target cells. Luciferase activity was measured in an Envision 2103 microplate luminometer.

### Virion-sACE2 Pull-Down Assay

Different amounts (1, 0.5 and 0.1 μg) of sACE2 was incubated with 30 μl of nickel-nitrilotriacetic acid-agarose beads (QIAGEN). After 1 hour of incubation, beads were washed with PBS, and 30µL of virus was added. Post 1 hour of incubation, the beads were washed, and bound protein was eluted with Laemmli loading buffer. The proteins were analyzed on an immunoblot probed with anti-p24 mAb (AG3.0) and horseradish peroxidase (HRP)-conjugated goat anti-mouse IgG secondary antibody (Sigma-Aldrich).

### Spike-sACE2-Nluc Binding Assay

HEK293T cells were transfected with variants spike expression vectors by lipofectamine 2000 (Invitrogen). 2.5 μg purified sACE2-Nuc protein prepared from transfected CHO cells was incubated with variant spikes-expressing cells for 30 minutes. Free sACE2-Nuc protein was washed away with PBS twice and luciferase substrates were added to the cells. Luciferase activity was measured using Nano-Glo luciferase substrate (Nanolight) in an Envision 2103 microplate luminometer (PerkinElmer).

### Immunoblot Analysis

Cells were lysed in buffer containing 50 mM HEPES, 150 mM KCl, 2 mM EDTA, 0.5% NP-40, and protease inhibitor cocktail. Protein (40 μg) was separated by SDS-PAGE. The proteins were transferred to polyvinylidene difluoride membranes and probed with anti-spike mAb (1A9) (GeneTex), anti-p24 mAb (AG3.0) and anti-GAPDH mAb (Life Technologies) followed by goat anti-mouse HRP-conjugated secondary antibody (Sigma). The proteins were visualized by luminescent substrate (Millipore) on iBright CL1000.

### Statistical Analysis

All experiments were in technical duplicates or triplicates. All plots and statistical analysis were performed on the mean value across technical duplicates. Statistical significance was determined by Wilcoxon non-parametric test and compared p values from the unpaired t test with confidence intervals shown as the mean ± SD or SEM. (*P ≤ 0.05, **P ≤ 0.01, ***P ≤ 0.001, ****P ≤ 0.0001). Spike protein structure (7BNM) (13) was downloaded from the Protein Data Bank.

## Results

### Variant Spike Protein Pseudotyped Lentiviruses

To analyze neutralizing antibodies against SARS-CoV-2 variants, we generated lentiviruses pseudotyped by the variant spike proteins. We previously reported on the production of Alpha, Beta, Gamma and Delta spike protein pseudotypes and have now added Delta plus and Lambda variant pseudotypes and spike proteins with the individual constituent mutations. The Delta and Delta Plus spike proteins have L452R and T478K mutations in the RBD (**Supplementary Figure 1A**); the Delta Plus spike protein contains an additional, K417N mutation in the RBD; and the Lambda spike protein has novel L452Q and F490S mutations in the RBD (**Supplementary Figure 1A**). The variant spike proteins were expressed well, proteolytically processed and incorporated into lentiviral virions at a level similar to that of the parental D614G spike protein in the producer cells and virions (**Supplementary Figure 1B**). The measurement of neutralizing antibody titers with such pseudotypes has been shown to yield results consistent with those obtained with the live virus plaque reduction neutralization test (14).

### Reduced Sensitivity of Virus With Variant Spikes to Neutralization by Convalescent Sera and mRNA Vaccine-Elicited Antibodies

**Figure 1:**
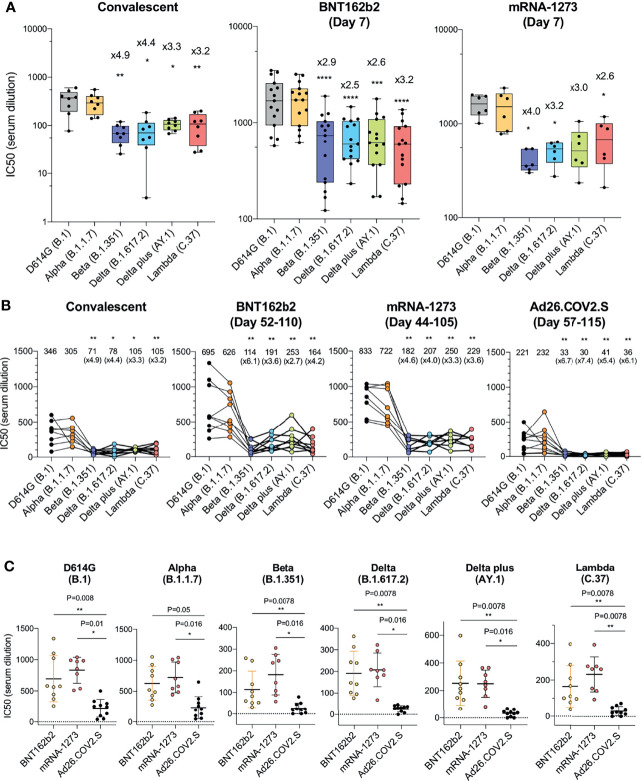
Comparison of neutralization titers of variant spike protein pseudotyped viruses by convalescent sera, antibodies elicited by BNT162b2, mRNA-1273, Ad26.COV2.S. **(A)** Neutralization of variant spike protein pseudotyped viruses by convalescent serum (n = 8) (left). Neutralizing titers of serum samples from BNT162b2 vaccinated individuals (n = 15) (middle). Neutralizing titers of serum samples from mRNA-1273 vaccinated donors (n = 6) (right). The serum was collected at early time point (7 days after second immunization). Serum samples from Ad26.COV2.S vaccinated donors were not available. The neutralization IC_50_ from individual donors is shown. Significance is based on two-sided t-test. Units on the Y axis of the graph for convalescent serum samples has been adjusted to allow for visualization of the data. **(B)** Comparison of neutralization of variants by convalescent serum (n = 8, the same donors in A), BNT162b2 vaccinated individuals (n = 9), mRNA-1273 vaccinated donors (n = 8), Ad26.COV2.S vaccinated donors (n = 10), sera from vaccinated individuals were collected at later time points [Day 52-110 (mean 90), Day 44-105 (mean 80), Day 57-115 (mean 82 days) after last immunization of each vaccine, see the **Supplementary Table 2**]. Each line shows individual donors. **(C)** Comparison of neutralization potency of each vaccine by different SARS-CoV-2 variants. The neutralization IC_50_ from individual donors vaccinated by BNT162b2 (yellow), mRNA-1273 (pink), Ad26.COV2.S (black) is shown. Significance is based on two-sided t-test. (*P ≤ 0.05, **P ≤ 0.01, ***P ≤ 0.001, ****P ≤ 0.0001).

Sera from individuals who had been infected prior to the emergence of the variants (collected 32-57 days post symptom onset) neutralized virus with the D614G spike protein with an average IC_50_ titer of 346 and neutralized the Alpha variant with a similar titer (IC_50_ of 305). Neutralizing titers for Beta, Delta, Delta plus and Lambda variants were decreased 3.2-4.9-fold relative to D614G, indicative of a modest resistance to neutralization (**Figure 1A** and **Supplementary Table 1**). The sera of individuals vaccinated with BNT162b2 and mRNA-1273 that were collected 7-days post-second injection – a peak antibody response timepoint - neutralized virus with the D614G spike with significantly higher titer (1835 and 1594, respectively) relative to the convalescent sera, and the antibodies cross-reacted on the variants with a modest 2.5-4.0-fold decrease in titer (**Figure 1A**). Analysis of the pseudotypes with single point mutations in the spike, neutralization resistance of the Beta variant can be attributed to the E484K mutation and neutralization of Delta by the L425R mutation. (**Supplementary Figures 2A–C**). Neutralization resistance of the lambda variant was attributed to the combined effect of the L452Q and F490S mutations (**Supplementary Figures 2A–C**).

### Partial Resistance of Viruses With Variant Spike Proteins to Neutralization by Ad26.COV2.S-Elicited Antibodies

We next compared the neutralizing titers of antibodies elicited by the BNT162b2 and mRNA-1273 mRNA vaccines with that of the Ad26.COV2.S adenoviral vector-based vaccine. The sera analyzed were collected from individuals at similar time-points post-final injection, (a mean of 90 days for BNT162b2, 80 day mean for mRNA-1273 and 82 day mean for Ad26.COV2.S; **Supplementary Table 2**) and from individuals of similar age and with similar clinical co-morbidities (**Supplementary Table 2**). None of the participants had a history of COVID-19 pre- or post-vaccination and all were negative for antibodies against the SARS-CoV-2 N protein (**Supplementary Table 2**). The results showed that BNT162b2 sera neutralized virus with the D614G and Alpha spikes with an average titer of 695 and 626. Compared to the D614G, the neutralizing titer against Beta was decreased 6.1-fold and Delta plus was decreased 2.7-fold. Results for the mRNA-1273 vaccine were similar with a 3.3-fold decrease in neutralizing titer for Delta plus and 4.6-fold for Beta. Ad26.COV2.S sera neutralized D614G and Alpha variants with average IC_50_ titers of 221 and 232, respectively, and neutralized the variants with titers that were decreased by 5.4-fold for Delta plus to 6.7-fold for the Beta variant as compared to D614G (**Figure 1B**). Presentation of the data grouped by variant shows decreased neutralizing titers against the variants by sera of the Ad26.COV2.S-vaccinated individuals (**Figure 1C**).

### Neutralization by REGN10933 and REGN10987

**Figure 2:**
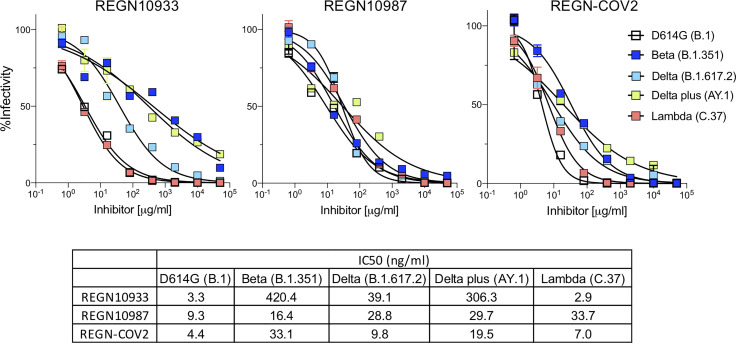
Neutralization of variant spike protein pseudotyped viruses by Regeneron antibodies. Neutralization of Beta, Delta, Delta plus and Lambda variant spike protein variants by REGN10933 and REGN10987 monoclonal antibodies. Neutralization of D614G and variant pseudotyped viruses by REGN10933 (left), REGN10987 (middle), and 1:1 ratio of REGN10933 and REGN10987 (right). The IC_50_ values of REGN10933, REGN10987 and the cocktail is shown in the table.

Analysis of REGN10933 and REGN10987 monoclonal antibodies that constitute the REGN-COV2 therapy showed that REGN10933 had decreased activity against the Beta variant spike which resulted in a 127-fold decrease in neutralizing titer. REGN10933 also had decreased activity against the Delta plus variant which resulted in a 92.7-fold decrease in neutralizing titer. The resistance to REGN10933 was attributed to K417N and E484K (**Supplementary Figure 2D**). REGN10933 neutralized virus with the Delta variant spike with a 12-fold decrease in titer which had only a minor effect on the activity of the cocktail. REGN10987 showed a minor reduction in neutralizing titer of virus with the Beta, Delta, Delta plus and Lambda variant spikes but this had little effect on neutralization of the virus by the cocktail (**Figure 2**). The resistance of variants to REGN10987 was attributed to the L452R/Q (**Supplementary Figure 2D**).

### The L452R/Q Mutation of the Delta Plus and Lambda Spike Proteins Increases Infectivity and Affinity for ACE2

**Figure 3:**
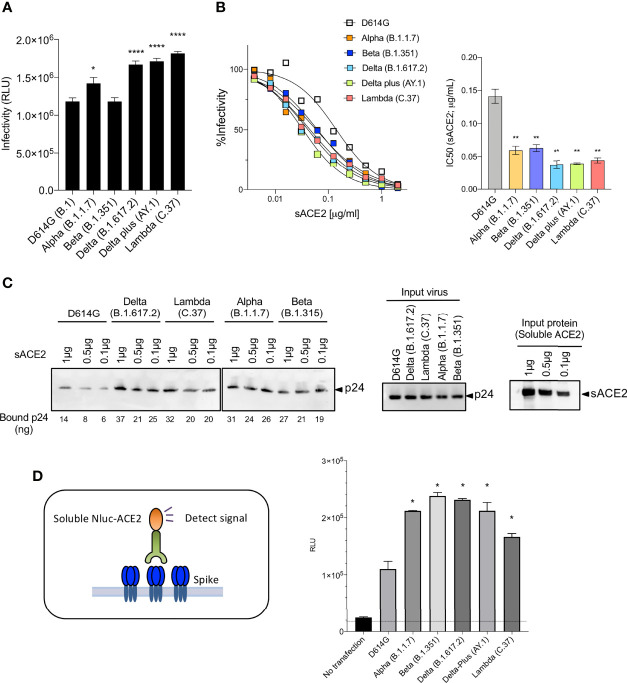
Neutralization of variant spike protein pseudotyped viruses by sACE2, monoclonal antibodies and virus-ACE2 binding assay. **(A)** Infectivity of virus pseudotyped by variant and D614G spike proteins. Viruses were normalized for RT activity and applied to target cells. Infectivity of viruses pseudotyped with the variant spike proteins were tested on ACE2.293T. Luciferase activity was measured two days post-infection. Significance was based on two-sided t-test. **(B)** Neutralization of variant spike protein variants by sACE2. Viruses pseudotyped with variant spike proteins were incubated with a serially diluted recombinant sACE2 and then applied to ACE2.293T cells. Each plot represents the percent infectivity of D614G and other variant spike pseudotyped viruses. The diagram shows the IC_50_ for each curve. **(C)** Nickel beads were coated for 1 hour with 1, 0.5 and 0.1 μg of sACE2 proteins. Unbound protein was removed and SARS-CoV-2 variant pseudotyped virions (D614G, Alpha, Beta, Delta, Lambda) were incubated with the beads. After 1 hour, the bound virions were analyzed on an immunoblot with antibody p24 antibody. Beads-bound p24 (ng) was calculated and indicated in the bottom (left). Input virions were analyzed on an immunoblot with anti-p24 antibody (middle). Input sACE2 proteins were analyzed on an immunoblot with anti-His-tag antibody (right). **(D)** Same amount (4μg) of spike was transfected on 2x10^6^ cells of 293T cells. Spike expressing cells were incubated with 0.2μg of sACE2-Nluc protein. After 30min of incubation, Luc activity was measured. (*P ≤ 0.05, **P ≤ 0.01, ****P ≤ 0.0001).

Measurement of the infectivity of the pseudotyped viruses, normalized for particle number, showed that the Lambda variant spike protein increased viral infectivity by 2-fold (**Figure 3A**), an increase equivalent to that of the Delta and Delta plus variants. The increase was due to the L452Q mutation and was similar to that of the L452R found in the Delta and Delta plus variants. The other mutations (Δ246-252, G75V-T76I, F490S and T859N) had no significant effect on infectivity (**Supplementary Figure 2E**). Measurement of the relative avidity of the variant spike proteins for ACE2 using sACE2 neutralization assay showed that variant spikes had a 3-fold increase in sACE2 avidity (**Figure 3B**). This increase was confirmed in a virion:ACE2 binding assay (**Figures 3C** and **Supplementary Figure 2F**) and spike:sACE2 binding assay (**Figure 3D**). The increase was caused by the L452R and L452Q mutation and were similar to the increase caused by the N501Y mutation (15, 16).

## Discussion

Previously identified SARS-CoV-2 variants have been found to be partially resistant to neutralization by vaccine-elicited antibodies (4–11). The data reported here extend those findings to the Delta plus and Lambda variants, both of which were resistant to mRNA vaccine-elicited antibodies to an extent similar to that of Beta and Delta. In sera collected ~3 months post-second immunization, BNT162b2 and mRNA-1273 mRNA vaccine-elicited antibodies neutralized the variants with a modest 3-fold average decrease in titer resulting in an average IC_50_ of about 1:600, a titer that is greater than that of convalescent sera and likely, a titer that in combination with post-vaccination T- and B-cell memory responses should provide durable protection against infection and disease.

Neutralizing antibodies elicited by Ad26.COV2.S vaccination had a significant decrease in neutralizing titer against the variants resulting in IC_50_ titers against Beta, Delta, Delta plus and Lambda variants that decreased 5-7-fold with mean neutralizing antibody titers of 33, 30, 41, and 36 against Beta, Delta, Delta plus and Lambda variant spikes, respectively. Modeling predicts that 50% protection from infection is provided by a titer that is 20% that of the mean convalescent titer (17). In this study, given a mean convalescent titer of 346 (**Supplementary Table 1**), 50% protection would correspond to an IC_50_ of 69. The titer required to protect against severe disease was shown to be 3% that of the mean titer of convalescent sera which in this study corresponds to a titer of 10. In a published report of phase 3 trial data, a single dose of Ad26.COV2.S, 28 days post administration, provided 64.0% protection against moderate to severe disease and 81.7% against severe-critical COVID-19 in South Africa where 95% of circulating SARS-CoV-2 was the Beta variant (18).

The data reported here differed somewhat from earlier reports in which Ad26.COV2.S-elicited antibody titers were maintained against the variants (19, 20) or decreased by 5-fold in neutralizing antibody titer against Beta and 3.3-fold decrease against Gamma (18). The source of such differences is unclear as those studies used a similar pseudotype assay to measure antibody neutralization and analyzed sera collected at a similar time-points post-immunization. Differences in experimental methods could account for the differences or the inclusion of some study participants with a history of previous infection.

Recent studies have shown that boosting a single immunization of the ChAdOx1nCoV-19 adenoviral vector vaccine with BNT162b2 resulted in high neutralizing titer against the VOCs (21–23). It is likely that neutralizing antibody titers against the VOCs elicited by the single shot Ad26.COV2.S could similarly be increased by a heterologous boost with an mRNA or alternative vaccine to avoid interference from of anti-adenovirus antibody induced by the earlier immunization. The continued protection afforded by Ad26.COV2.S vaccination despite decreased neutralizing antibody against the variants suggest a role for non-neutralizing antibodies mediated by the immunoglobulin Fc region (18) and a role for T cells elicited by vaccination. While the single dose vaccination provides a high degree of protection, a second immunization, particularly for those with a poor response to the initial injection or who are immunocompromised, could be valuable.

In our study, one subject who received Ad26.COV2.S vaccine was found to have a previous history of SARS-CoV-2 infection as determined by anti-N antibody serum titer. This donor was excluded from the study, but interestingly, the serum neutralizing antibody titers from this study participant were higher than any of the other sera tested all of which were donated by inexperienced study participants (IC50 3177 against D614G, 760 against Beta, 1641 against Delta, 1510 against Delta plus and 1167 against Lambda; data not shown). The is only a single individual and therefore not statistically significant but raises the possibility that the previous infection results in a stronger response to vaccination.

The variant spike proteins resulted in increased infectivity of the pseudotyped viruses compared to the parental D614G spike protein, consistent with the high transmissibility of the viruses in human populations. The increased infectivity appeared to result from an increased affinity of the variant spike proteins for ACE2. Alpha, Beta, Delta and Delta plus spike proteins had similarly high affinity for ACE2, consistent with earlier reports (15, 16) while Lambda was intermediate in affinity. The increases in affinity mapped to the N501Y mutation in Beta and the L452R and L452Q mutations in Delta and Delta plus, consistent with previous reports (24, 25). The effects of the mutations of amino acid 452 are not clear as the residue does lie within the site of interaction with ACE2 but is located in a negatively charged patch; the positive charge introduced by the L452R mutation may serve to increase electrostatic complementarity. Mutation of amino acid 452 could act by altering the conformation of the interaction domain of the spike protein.

The therapeutic monoclonal antibodies that have been highly successful in the treatment of COVID-19 largely maintained their ability to neutralize viruses with the variant spike proteins. REGN10987 maintained its neutralizing titer against the Alpha, Beta, Delta, Delta plus and Lambda variants while REGN10933 lost neutralizing activity against Beta and Delta plus, effects that were caused by the E484K and K417N mutations in Beta and Delta plus, consistent an earlier report (5). L452R of Delta and Delta plus also caused a small decrease in the neutralizing titers of REGN10933 and REGN10987 as did the L452Q mutation in the Lambda spike protein. Despite the partial loss of activity of the single monoclonal antibodies, the neutralizing activity of the antibody cocktail remained high.

Our findings emphasize the importance of surveillance for breakthrough infections with the highly transmissible variants. An increased frequency of breakthrough infections accompanied by severe COVID-19 following vaccination would provide a rationale for booster immunizations to protect against the current and future variants that may arise. The recent appearance of the very highly mutated Omicron virus raises the possibility that the virus be even further resistant to vaccine-elicited antibodies and will result in a high frequency of breakthrough infection. While recent data suggest that a second immunization results in an anamnestic rise in circulating antibody levels, the public health apparatus should maintain its focus on primary immunization in the U.S. and globally.

## Study Limitations

This study was performed with relatively small number of participants who enrolled in the clinical studies at the NYULH Vaccine Center which could limit the resolution of differences in antibody titers. The sample number used was would have achieved a higher confidence level with the larger sample numbers. The study also did not evaluate effects of participant age or at high risk of disease. The sera samples were collected in single time point which does not provide insight into the durability of the antibody titers.

## Data Availability Statement

The raw data supporting the conclusions of this article will be made available by the authors, without undue reservation.

## Ethics Statement

The clinical study was conducted at the NYU Vaccine Center with participant’s written consent under IRB-approved protocols (18-02035 and 18-02037). The patients/participants provided their written informed consent to participate in this study.

## Author Contributions

TT and NL designed the experiments. MS, AC, RH, and MM supervised specimen selection and the collection of clinical information, did the ELISAs and provided reagents and key insights. TT, HZ, and BD did the *in vitro* analyses. TT, HZ, MM, and NL wrote the manuscript. All authors contributed to the article and approved the submitted version.

## Funding

The work was funded in part by grants from the NIH to NL (DA046100, AI122390 and AI120898) and to MM (UM1AI148574). TT was supported by the Vilcek/Goldfarb Fellowship Endowment Fund. MM and MS were partially supported by NYU Grossman School of Medicine institutional support.

## Conflict of Interest

MM received research grants from Lilly, Pfizer, and Sanofi, and serves on advisory boards for Pfizer, Merck, and Meissa Vaccines.

The remaining authors declare that the research was conducted in the absence of any commercial or financial relationships that could be construed as a potential conflict of interest.

## Publisher’s Note

All claims expressed in this article are solely those of the authors and do not necessarily represent those of their affiliated organizations, or those of the publisher, the editors and the reviewers. Any product that may be evaluated in this article, or claim that may be made by its manufacturer, is not guaranteed or endorsed by the publisher.
