# Supplementary material for: Neutralization of SARS-CoV-2 Variants by mRNA and Adenoviral Vector Vaccine-Elicited Antibodies
Source: Front Immunol. 2022 Mar 8;13:797589. doi: 10.3389/fimmu.2022.797589 (PMC8957851; doi:10.3389/fimmu.2022.797589)
Supplement: Supplementary file 1 [file DataSheet_1.docx]

**Supplementary Table 1**. Neutralization of variants by convalescent sera, BNT162b2 and mRNA-1273 elicited antibodies 7 days post-second vaccination.

|  | **Convalescent (32-57 days post-symptom onset)** | | | | | |
| --- | --- | --- | --- | --- | --- | --- |
|  | IC_50_(serum dilution) | | | | | |
| donor | D614G  (B.1) | Alpha (B.1.1.7) | Beta (B.1.351) | Delta (B.1.617.2) | Delta plus  (AY.1) | Lambda  (C.37) |
| 1 | 251 | 312 | 69 | 56 | 112 | 94 |
| 2 | 176 | 223 | 91 | 84 | 121 | 124 |
| 3 | 77 | 140 | 68 | 185 | 132 | 29 |
| 4 | 406 | 375 | 38 | 51 | 68 | 121 |
| 5 | 602 | 146 | 57 | 3 | 77 | 27 |
| 6 | 383 | 416 | 119 | 34 | 142 | 199 |
| 7 | 520 | 556 | 100 | 119 | 89 | 187 |
| 8 | 359 | 273 | 25 | 94 | 100 | 61 |
| Mean (SD) | 346  (174) | 305  (142) | 71  (32) | 78  (56) | 105  (26) | 105  (66) |

|  |  |  |  | **BNT162b2** | | | | | |
| --- | --- | --- | --- | --- | --- | --- | --- | --- | --- |
|  |  |  |  | IC_50_(serum dilution) | | | | | |
| donor | Days post last vaccine | sex | age | D614G  (B.1) | Alpha (B.1.1.7) | Beta (B.1.351) | Delta (B.1.617.2) | Delta plus  (AY.1) | Lambda  (C.37) |
| 1 | 7 | M | 33 | 1915 | 1994 | 877 | 914 | 575 | 834 |
| 2 | 7 | M | 39 | 697 | 615 | 228 | 231 | 169 | 191 |
| 3 | 7 | F | 29 | 2572 | 2026 | 1366 | 950 | 1088 | 1244 |
| 4 | 7 | F | 37 | 939 | 925 | 145 | 507 | 171 | 123 |
| 5 | 7 | M | 43 | 1445 | 1717 | 161 | 416 | 361 | 167 |
| 6 | 7 | F | 39 | 2205 | 2069 | 413 | 370 | 614 | 935 |
| 7 | 7 | M | 65 | 1689 | 1259 | 918 | 560 | 1769 | 735 |
| 8 | 7 | M | 50 | 3189 | 2676 | 1045 | 1095 | 762 | 1032 |
| 9 | 7 | F | 21 | 1352 | 1720 | 456 | 594 | 363 | 451 |
| 10 | 7 | M | 49 | 1170 | 1355 | 604 | 669 | 796 | 635 |
| 11 | 7 | M | 22 | 672 | 729 | 219 | 398 | 592 | 238 |
| 12 | 7 | F | 39 | 571 | 841 | 364 | 441 | 480 | 259 |
| 13 | 7 | M | 50 | 3338 | 3099 | 1245 | 1463 | 1241 | 926 |
| 14 | 7 | M | 55 | 3486 | 3181 | 591 | 1042 | 685 | 1200 |
| 15 | 7 | F | 36 | 2294 | 2257 | 654 | 1092 | 1138 | 1888 |
| Mean  (SD) |  |  | 40 | 1835  (986) | 1764  (822) | 619  (394) | 716  (354) | 720  (436) | 724  (502) |

|  |  |  |  | **mRNA-1273** | | | | | |
| --- | --- | --- | --- | --- | --- | --- | --- | --- | --- |
|  |  |  |  | IC_50_(serum dilution) | | | | | |
| donor | Days post last vaccine | sex | age | D614G  (B.1) | Alpha (B.1.1.7) | Beta (B.1.351) | Delta (B.1.617.2) | Delta plus  (AY.1) | Lambda  (C.37) |
| 1 | 7 | F | 44 | 1380 | 1186 | 532 | 500 | 382 | 472 |
| 2 | 7 | F | 32 | 1963 | 1852 | 362 | 614 | 731 | 1185 |
| 3 | 7 | F | 52 | 1010 | 833 | 351 | 273 | 1055 | 209 |
| 4 | 7 | M | 65 | 1305 | 779 | 298 | 419 | 234 | 427 |
| 5 | 7 | M | 55 | 1879 | 2395 | 535 | 638 | 411 | 880 |
| 6 | 7 | M | 50 | 2028 | 1990 | 322 | 568 | 615 | 946 |
| Mean  (SD) |  |  | 50 | 1594  (419) | 1506  (668) | 400  (106) | 502  (138) | 571  (296) | 687  (373) |

**Supplementary Table 2.** Neutralization of viruses by sera from BNT162b2, mRNA-1273 and Ad26.COV.S vaccinated individuals.

| **BNT162b2 (52-110 days post last vaccine: mean 90)** | | | | | | | | | | | |
| --- | --- | --- | --- | --- | --- | --- | --- | --- | --- | --- | --- |
|  |  |  |  |  |  | IC_50_(serum dilution) | | | | | |
| donor | Days post last vaccine | Anti-N ELISA | Age | Sex | Comorbidities | D614G | Alpha | Beta | Delta | Delta plus | Lambda |
| 1 | 84 | - | 39 | F | None | 575 | 427 | 51 | 141 | 215 | 167 |
| 2 | 52 | - | 23 | F | None | 1338 | 1055 | 82 | 314 | 296 | 101 |
| 3 | 101 | - | 26 | F | Asthma | 1101 | 829 | 258 | 362 | 598 | 209 |
| 4 | 109 | - | 33 | F | None | 562 | 750 | 138 | 243 | 186 | 111 |
| 5 | 60 | - | 35 | F | Hypothyroidism, Psoriasis | 1024 | 930 | 53 | 239 | 391 | 284 |
| 6 | 81 | - | 42 | F | Asthma | 258 | 279 | 32 | 103 | 248 | 39 |
| 7 | 108 | - | 26 | F | None | 580 | 485 | 247 | 95 | 133 | 396 |
| 8 | 107 | - | 24 | M | None | 372 | 520 | 104 | 77 | 147 | 78 |
| 9 | 110 | - | 35 | M | None | 445 | 362 | 60 | 148 | 67 | 95 |
| Mean (SD) | 90  (22) |  | 31 |  |  | 695  (369) | 626  (272) | 114  (85) | 191  (102) | 253  (161) | 164  (114) |

| **mRNA-1273 (44-105 days post last vaccine: mean 80)** | | | | | | | | | | | |
| --- | --- | --- | --- | --- | --- | --- | --- | --- | --- | --- | --- |
|  |  |  |  |  |  | IC_50_(serum dilution) | | | | | |
| donor | Days post last vaccine | Anti-N ELISA | Age | Sex | Comorbidities | D614G | Alpha | Beta | Delta | Delta plus | Lambda |
| 1 | 89 | - | 26 | M | None | 984 | 1043 | 108 | 173 | 364 | 257 |
| 2 | 92 | - | 53 | M | None | 972 | 703 | 237 | 207 | 239 | 273 |
| 3 | 61 | - | 67 | M | Prediabetes | 774 | 544 | 87 | 68 | 264 | 139 |
| 4 | 93 | - | 33 | F | None | 509 | 443 | 58 | 209 | 82 | 91 |
| 5 | 44 | - | 32 | M | None | 856 | 579 | 273 | 203 | 365 | 258 |
| 6 | 100 | - | 29 | F | None | 1038 | 1014 | 305 | 295 | 312 | 274 |
| 7 | 52 | - | 33 | F | None | 990 | 968 | 145 | 322 | 213 | 152 |
| 8 | 105 | - | 55 | F | Asthma | 537 | 485 | 246 | 184 | 160 | 391 |
| Mean (SD) | 80  (24) |  | 41 |  |  | 833  (209) | 722  (249) | 182  (94) | 208  (77) | 250  (99) | 229  (96) |

| **Ad26.COV2.S**  (**57-115 days** **post last vaccine: mean 82)** | | | | | | | | | | | |
| --- | --- | --- | --- | --- | --- | --- | --- | --- | --- | --- | --- |
|  |  |  |  |  |  | IC_50_(serum dilution) | | | | | |
| donor | Days post last vaccine | Anti-N ELISA | Age | Sex | Comorbidities | D614G | Alpha | Beta | Delta | Delta plus | Lambda |
| 1 | 57 | - | 42 | F | None | 46 | 55 | 22 | 31 | 41 | 21 |
| 2 | 58 | - | 28 | F | None | 133 | 101 | 5 | 28 | 46 | 47 |
| 3 | 66 | - | 36 | F | None | 500 | 130 | ND | ND | ND | ND |
| 4 | 92 | - | 33 | F | None | 333 | 257 | 23 | 31 | 8 | 24 |
| 5 | 87 | - | 39 | F | Prediabetes | 244 | 205 | 19 | 42 | 31 | 36 |
| 6 | 72 | - | 32 | M | None | 268 | 308 | 79 | 34 | 63 | 59 |
| 7 | 92 | - | 39 | F | None | 251 | 377 | 44 | 46 | 38 | 70 |
| 8 | 71 | - | 75 | F | None | 298 | 648 | ND | 7 | ND | ND |
| 9 | 105 | - | 30 | M | None | 38 | 45 | 18 | 37 | 31 | 13 |
| 10 | 115 | - | 33 | F | None | 98 | 194 | 50 | 15 | 68 | 20 |
| Mean (SD) | 82  (20) |  | 39 |  |  | 221  (144) | 232  (182) | 33  (24) | 30  (12) | 41  (19) | 36  (21) |


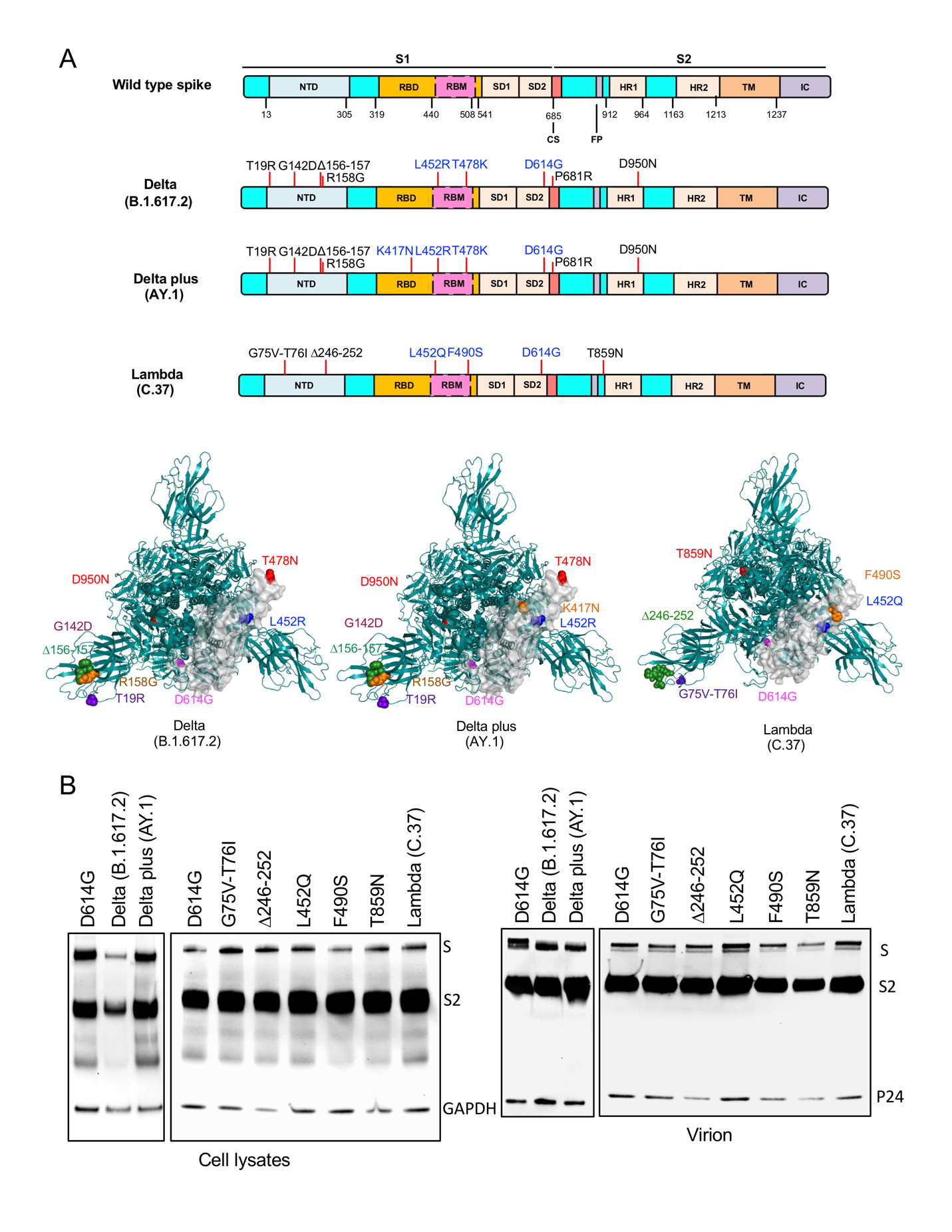


**Supplementary Figure 1.**

**The structure of variant spikes and immunoblot analysis of spike proteins.**

(A) The domain structure of the SARS-CoV-2 spike is diagrammed with Delta (B.1.617.2), Delta plus (AY.1), Lambda (C.37) variant amino acid residues indicated. NTD, N-terminal domain; RBD, receptor-binding domain; RBM, receptor-binding motif; SD1 subdomain 1; SD2, subdomain 2; CS, cleavage site; FP, fusion peptide; HR1, heptad repeat 1; HR2, heptad repeat 2; TM, transmembrane region; IC, intracellular domain. Key mutations are shown in 3D structure (top view).

(B) Immunoblot analysis of the Delta (B.1.617.2), Delta plus (AY.1), single point mutated of Lambda (C.37) variant, Lambda (C.37) variant spike proteins in transfected 293T cells. Pseudotyped viruses were produced by transfection of 293T cells. Two days post-transfection, virions were analyzed on an immunoblot probed with anti-spike antibody and anti-HIV-1 p24. The cell lysates were probed with anti-spike antibody and anti-GAPDH antibodies as a loading control.

**Supplementary Figure 2.**

**Neutralization titers of spike protein pseudotyped viruses (single point mutations) by convalescent sera, antibodies elicited by BNT162b2, mRNA-1273.**

(A) Neutralization of variant spike protein (single point mutations) pseudotyped viruses by convalescent serum (n=8). Dots represent the IC_50_ of single donors.

(B) Neutralizing titers of serum samples from BNT162b2 vaccinated individuals (n=15). The serum was collected at early time point (7 days after second immunization). Each dot represents the IC_50_ for a single donor.

(C) Neutralizing titers of serum samples from mRNA-1273 vaccinated donors (n=6). The serum was collected at early time point (7 days after second immunization). The neutralization IC_50_ from individual donors is shown. Significance is based on the two-sided t-test.

(D) Neutralization of variant spike protein variants (single point mutations) by REGN10933 and REGN10987 monoclonal antibodies. The IC_50_ of REGN10933, REGN10987 and the cocktail is shown in the table.

(E) Infectivity of pseudotyped virus. Viruses were normalized for RT activity and applied to target cells. Infectivity of viruses pseudotyped with the individual variant mutations were tested on ACE2.293T. Luciferase activity was measured two days post-infection. Significance was based on two-sided t-test.

(F) Neutralization of variant spike protein variants by sACE2. Viruses pseudotyped with individual variant mutations were incubated with a serially diluted recombinant sACE2 and then applied to ACE2.293T cells. The diagram shows the IC_50_ for each curve.
